# Laboratory diagnostics of murine blood for detection of mouse cytomegalovirus (MCMV)-induced hepatitis

**DOI:** 10.1038/s41598-018-33167-7

**Published:** 2018-10-04

**Authors:** Felix R. Stahl, Roman Jung, Virginija Jazbutyte, Eléonore Ostermann, Silvia Tödter, Renke Brixel, Annette Kemmer, Stephan Halle, Stefan Rose-John, Martin Messerle, Petra C. Arck, Wolfram Brune, Thomas Renné

**Affiliations:** 1 Institute of Clinical Chemistry and Laboratory Medicine, University Medical Center Hamburg-Eppendorf, Hamburg, Germany; 2 Heinrich Pette Institute, Leibniz Institute for Experimental Virology, Hamburg, Germany; 3 German Center for Infection Research (DZIF), partner site Hamburg, Hamburg, Germany; 4 Institute of Immunology, Hannover Medical School, Hannover, Germany; 5 Institute of Biochemistry, University of Kiel, Kiel, Germany; 6 Institute of Virology, Hannover Medical School, Hannover, Germany; 7 Department of Obstetrics and Fetal Medicine, University Medical Center Hamburg-Eppendorf, Hamburg, Germany

## Abstract

Mouse models are important and versatile tools to study mechanisms and novel therapies of human disease *in vivo*. Both, the number and the complexity of murine models are constantly increasing and modification of genes of interest as well as any exogenous challenge may lead to unanticipated biological effects. Laboratory diagnostics of blood samples provide a comprehensive and rapid screening for multiple organ function and are fundamental to detect human disease. Here, we adapt an array of laboratory medicine-based tests commonly used in humans to establish a platform for standardized, multi-parametric, and quality-controlled diagnostics of murine blood samples. We determined sex-dependent reference intervals of 51 commonly used laboratory medicine tests for samples obtained from the C57BL/6J mouse strain. As a proof of principle, we applied these diagnostic tests in a mouse cytomegalovirus (MCMV) infection model to screen for organ damage. Consistent with histopathological findings, plasma concentrations of liver-specific enzymes were elevated, supporting the diagnosis of a virus-induced hepatitis. Plasma activities of aminotransferases correlated with viral loads in livers at various days after MCMV infection and discriminated infected from non-infected animals. This study provides murine blood reference intervals of common laboratory medicine parameters and illustrates the use of these tests for diagnosis of infectious disease in experimental animals.

## Introduction

Since the 1980s, the use of mouse models for research in life science has increased continuously. Currently, the number of documented mouse genotypes with phenotype annotation reaches almost 60000^1^. Genetic modification of mice allows for modeling of various human diseases and targeted deletion of a specific gene of interest may help to unravel the underlying molecular mechanisms. However, any genetic modification may cause unpredicted consequences in organ systems that express the gene or are indirectly affected by the presence or absence of the gene product. Similarly, any challenge such as application of pharmacologic agents and microbes, or invasive surgery may lead to unexpected and complex effects. Thus, in addition to histopathology or additional cellular and molecular readout approaches, a comprehensive and rapid organ-specific assessment of newly generated mouse models is needed to identify such unanticipated effects and reduce the risk of assessing epiphenomena rather than the gene or intervention of interest.

Laboratory blood tests are commonly used in clinical pathology to detect organ damage by measurement of surrogate markers in body fluids of human patients. The majority of laboratory tests used for human samples are not applicable for measurement of murine samples. This is mainly due to the fact that most laboratory tests are based on immunoassays using antibodies that are species-specific and recognize a unique peptide sequence within the protein of interest. Thus, non-conserved amino acid sequences of human and mouse proteins as well as posttranslational modifications may interfere with cross-species binding of the antibodies used in the immunoassays. However, some antibodies bind to conserved epitopes, cross-react among various species and these immunoassays may be used to analyze both human and mouse samples. In addition to antibody-based immunoassays, determination of plasma electrolyte concentration using ion-sensitive electrodes and enzymatic activities of plasma proteins using chromogenic substrates are used in clinical chemistry tests with unknown species-specific sensitivities. Another critical issue is the sample volume needed for analysis of a single parameter. Some clinical chemistry analyzers used for human diagnostics require 250 µl of plasma per measurement. Given that a mouse with a body weight of 20 grams has an estimated total blood volume of 1500 µl the number of parameters to be analyzed per animal is restricted due to limited material. Accordingly, a rational selection of laboratory tests is required to detect and monitor pathological conditions in peripheral blood of mice.

Human blood reference intervals are well established for laboratory tests and needed for interpretation of a measured value. Reference intervals may depend on age, sex, ethnical background, pregnancy, medical treatment, or sampling conditions. Some are specific for the various analyzers, tests and reagents used and thus are provided by the manufacturer of the test. In contrast to human samples there is a lack of reference intervals for laboratory tests of murine samples. Thus, to utilize laboratory medicine tests for diagnosis of mouse samples reference intervals based on data from healthy animals need to be determined.

Human cytomegalovirus (HCMV) is a highly prevalent opportunistic pathogen that may cause disease in various organs such as pneumonitis, colitis, and hepatitis. HCMV infection and disease are important causes of morbidity and mortality in transplant recipients^2^. Moreover, congenital infection with HCMV is the most frequent cause of permanent disabilities in neonates^3^. However, the majority of immune-competent adults experience only mild or no symptoms after infection^4^. Cytomegaloviruses exhibit distinct species specificity and thus mice cannot be infected with HCMV. Though, infection of mice with the related mouse cytomegalovirus (MCMV) is a well-established model to study cytomegalovirus pathogenesis and antiviral immune responses *in vivo*^5,6^. In both, humans and mice, lymphocytes have been shown to be required for infection control and protection of the host from cytomegalovirus disease^7–11^. *Rag2*^−/−^*Il2rg*^−/−^ mice lack B, T and natural killer (NK) cells and thus provide a model to further study the role of lymphocytes in antiviral immunity.

Here, we established a panel of 51 laboratory tests including clinical chemistry-based parameters as well as a differential blood count for samples of C57BL/6J mice. We determined sex-dependent reference intervals for each parameter. Applying this panel in an infection model allowed for detection of MCMV-induced liver damage in mice. In line with elevated enzymatic activities of liver proteins in the blood, we found histopathological evidence of severe virus hepatitis. Moreover, all MCMV-infected animals had plasma activity of alanine aminotransferase (ALAT) above the upper limit of the reference interval determined from healthy animals. Accordingly, the use of laboratory medicine-based diagnostics of mouse blood allows for diagnosis of organ disease in experimental animals with versatile possible applications.

## Results

### Reference intervals and sex differences for laboratory diagnostic parameters of peripheral murine blood

To create a diagnostic screening panel that allows for detection of organ dysfunction we selected for 61 commonly used clinical chemistry parameters including plasma proteins, electrolytes, lipids, hormones, metabolites as well as a differential blood count that comprises additional 26 parameters. We analyzed peripheral blood obtained from inbred C57BL/6J mice. Notably, we found concentrations were below range of linearity of the analyzer or not detectable at all in 30 out of the selected 61 clinical chemistry parameters. Another six parameters required too large plasma volumes to allow for detailed analysis (Supplementary Information Table 1). However, 25 clinical chemistry parameters lead to reproducible signals using the tests and protocols that were designed for the analysis of human blood samples. In addition, 26 differential blood count parameters could be determined reliably utilizing an analyzer designed for veterinary blood samples. Thus, the final panel included a total of 51 diverse parameters that are indicative for functionality of various organs or pathophysiological processes (Tables 1 and 2). One analysis required approximately 300 µl of plasma for all clinical chemistry parameters and 50 µl of whole blood for a differential blood count. Thus, animals had to be exsanguinated to draw sufficient material for measurement of all 51 parameters at once. However, a smaller panel including seven screening clinical chemistry parameters (e.g. ALAT, creatinine, creatine kinase, cholesterol, lactate dehydrogenase (LDH), lipase, and total protein) can be processed with 100 µl of plasma, thus allowing for comprehensive diagnostics without sacrificing of the experimental mouse. Hence, the laboratory tests analyzed here allow for general screening of healthy and diseased mice but also for monitoring of specific organ damage.

**Table 1:** Reference intervals for clinical chemistry parameters.

Parameter	Unit	Pooled Reference interval (mean or *median*, samples)	Female Reference interval (mean or *median*, samples)	Male Reference interval (mean or *median*, samples)
ALAT	U/l	<54 (27, n = 40)	<71 (31, n = 17)	10–38 (23, n = 23)
albumin	g/l	11–15 (13, n = 40)	n/a (14, n = 23)	n/a (12, n = 17)
AP	U/l	154–292 (220, n = 49)	188–290 (239, n = 27)	135–256 (197, n = 22)
ASAT	U/l	2–90 (47, n = 64)	12–81 (47, n = 34)	0–102 (46, n = 30)
calcium	mmol/l	2.10–2.41 (2.25, n = 35)	2.08–2.33 (2.21, n = 17)	2.16–2.46 (2.29, n = 18)
CHE	U/ml	3.3–6.2 (4.8, n = 38)	4.3–6.2 (5.3, n = 21)	3.4–4.7 (4.1, n = 17)
chloride	mmol/l	107–119 (113, n = 35)	106–122 (114, n = 17)	107–118 (112, n = 18)
cholesterol	mg/dl	42–88 (66, n = 38)	48–67 (58, n = 21)	60–92 (76, n = 17)
creatinine	mg/dl	0.14–0.33 (0.24, n = 40)	0.15–0.32 (0.23, n = 23)	0.14–0.36 (0.25, n = 17)
creatine kinase	U/l	61–650 (344, n = 60)	55–640 (370, n = 36)	41–686 (367, n = 24)
fT3	pmol/l	3.4–5.8 (4.6, n = 40)	3.3–5.8 (4.5, n = 23)	3.3–6.0 (4.7, n = 17)
fT4	pmol/l	15.8–27.2 (*21.5*, n = 40)	16.0–27.2 (21.6, n = 23)	14.3–27.8 (*21.1*, n = 17)
ɣ-GT	U/l	6–9 (7, n = 40)	n/a (7, n = 23)	5–9 (7, n = 17)
GLDH	U/l	0–26.2 (*10.1*, n = 38)	2.9–14.1 (8.6, n = 23)	0–36.2 (*14.6*, n = 15)
glucose	mg/dl	237–507 (367, n = 37)	235–485 (355, n = 21)	225–552 (382, n = 16)
HDL	mg/dl	39–84 (62, n = 40)	43–68 (56, n = 23)	54–89 (71, n = 17)
iron	µmol/l	14.9–30.9 (22.8, n = 35)	17.6–34.1 (*26.1*, n = 17)	13.9–26.9 (20.8, n = 18)
LDH	U/l	52–304 (180, n = 73)	114–221 (167, n = 8)	19–357 (194, n = 35)
lipase	U/l	<323 (160, n = 40)	<369 (142, n = 23)	113–225 (170, n = 17)
magnesium	mmol/l	1.00–1.24 (1.12, n = 35)	0.97–1.23 (1.10, n = 17)	1.01–1.26 (1.13, n = 18)
potassium	mmol/l	2.8–4.4 (3.7, n = 35)	2.7–4.2 (3.5, n = 17)	3.1–4.6 (3.9, n = 18)
protein	g/l	40–47 (43, n = 40)	39–46 (43, n = 23)	41–46 (44, n = 17)
sodium	mmol/l	143–152 (147, n = 35)	143–153 (148, n = 17)	143–151 (147, n = 18)
triglycerides	mg/dl	31–151 (94, n = 40)	36–119 (79, n = 23)	53–174 (114, n = 17)
urea nitrogen	mg/dl	17–29 (23, n = 38)	15–27 (21, n = 21)	20–29 (24, n = 17)

ALAT, alanine aminotransferase; ASAT, aspartate aminotransferase; ɣ-GT, gamma-glutamyl transpeptidase; GLDH, glutamate dehydrogenase; LDH, lactate dehydrogenase; AP, alkaline phosphatase; CHE, pseudocholinesterase; fT3, free triiodothyronine; fT4, free thyroxine; HDL, high density lipoprotein. n/a = not applicable for albumin and ɣ-GT as plasma levels showed too low variance to perform statistics.

**Table 2:** Reference intervals for hematology parameters.

Parameter	Unit	Pooled Reference interval (mean or *median*, samples)	Female Reference interval (mean or *median*, samples)	Male Reference interval (mean or *median*, samples)
red blood cells	10^6^/µl	8.50–9.95 (9.3, n = 39)	8.48–10. 16 (9.3, n = 18)	8.46–9.81 (9.2, n = 21)
hemoglobin	g/dl	12.7–15.1 (14.0, n = 39)	12.9–15.7 (14.3, n = 18)	12.8–14.7 (13.8, n = 21)
hematocrit	%	44.3–53.1 (48.7, n = 39)	44.4–54.7 (49.4, n = 18)	44.2–51.9 (48.2, n = 21)
MCV	fl	50.2–55.3 (52.6, n = 39)	50.5–55.5 (52.9, n = 18)	49.7–55.3 (52.3, n = 21)
MCH	pg	14.7–15.6 (15.1, n = 39)	14.9–15.7 (15.3, n = 18)	14.6–15.4 (15.0, n = 21)
MCHC	g/dl	27.5–29.9 (28.8, n = 39)	27.8–29.8 (*28.8*, n = 18)	27.1–30.0 (28.7, n = 21)
RDW-CV	%	20.7–23.8 (*22.3*, n = 39)	20.3–24.2 (*22.3*, n = 18)	21.0–23.6 (22.3, n = 21)
RDW-SD	fl	27.1–33.5 (30.4, n = 39)	27.1–33.8 (30.7, n = 18)	26.7–33.4 (30.1, n = 21)
reticulocytes	10^3^/µl	256.0–511.1 (*386.9*, n = 39)	182.7–540.8 (*366.0*, n = 18)	314.2–477.7 (397.2, n = 21)
reticulocytes	%	2.75–5.54 (*4.18*, n = 39)	1.88–5.85 (*3.87*, n = 18)	3.46–5.14 (4.32, n = 21)
leukocytes	10^3^/µl	3.23–13.34 (7.90, n = 39)	3.35–12.32 (7.85, n = 18)	2.49–14.55 (7.94, n = 21)
lymphocytes	10^3^/µl	2.73–11.68 (6.86, n = 39)	2.82–10.92 (6.93, n = 18)	2.09–12.84 (6.81, n = 21)
neutrophils	10^3^/µl	0.03–1.12 (0.62, n = 39)	0.15–0.89 (0.52, n = 18)	0.03–1.34 (0.71, n = 21)
monocytes	10^3^/µl	0.00–0.54 (*0.25*, n = 39)	0.00–0.49 (*0.22*, n = 18)	0.00–0.64 (0.30, n = 21)
eosinophils	10^3^/µl	0.00–0.24 (0.12, n = 39)	0.02–0.24 (0.14, n = 18)	0.00–0.25 (0.12, n = 21)
basophils	10^3^/µl	n/a (*0.0*, n = 39)	n/a (*0.0*, n = 18)	n/a (*0.0*, n = 21)
lymphocytes	%	70.0–94.6(86.7, n = 39)	83.5–92.6 (88.2, n = 18)	77.0–94.3 (85.5, n = 21)
neutrophils	%	1.7–13.4 (*8.0*, n = 39)	3.4–10.0 (6.7, n = 18)	1.9–15.7 (9.3, n = 21)
monocytes	%	0.4–6.1 (*3.3*, n = 39)	0.3–5.7 (*2.9*, n = 18)	0.3–6.8 (3.8, n = 21)
eosinophils	%	0.5–2.5 (1.5, n = 39)	0.7–2.8 (1.7, n = 18)	0.4–2.3 (1.4, n = 21)
basophils	%	n/a (*0.0*, n = 39)	n/a (*0.0*, n = 18)	n/a (*0.0*, n = 21)
platelets	10^3^/µl	476–893 (684, n = 39)	505–692 (595, n = 18)	656–881 (767, n = 21)
plateletcrit	%	0.28–0.53 (0.40, n = 39)	0.30–0.42 (*0.36*, n = 18)	0.38–0.53 (0.45, n = 21)
MPV	fl	5.7–6.2 (5.9, n = 39)	5.7–6.2 (6.0, n = 18)	5.6–6.1 (5.9, n = 21)
PDW	fl	6.3–7.3 (6.8, n = 39)	6.3–7.3 (6.8, n = 18)	6.3–7.3 (6.8, n = 21)
P-LCR	%	1.3–4.0 (2.6, n = 39)	1.1–4.1 (2.6, n = 18)	1.4–3.9 (2.6, n = 21)

MCV, mean corpuscular volume; MCH, mean cellular hemoglobin; MCHC, mean corpuscular/cellular hemoglobin concentration; RDW-CV, red blood cell distribution width - coefficient of variation; RDW-SD, red blood cell distribution width - standard deviation; MPV, mean platelet volume; PDW, platelet distribution width; P-LCR, platelet large cell ratio. n/a = not applicable for basophils as the absolute numbers of events counted was too low to perform statistics.

**Figure 1:**
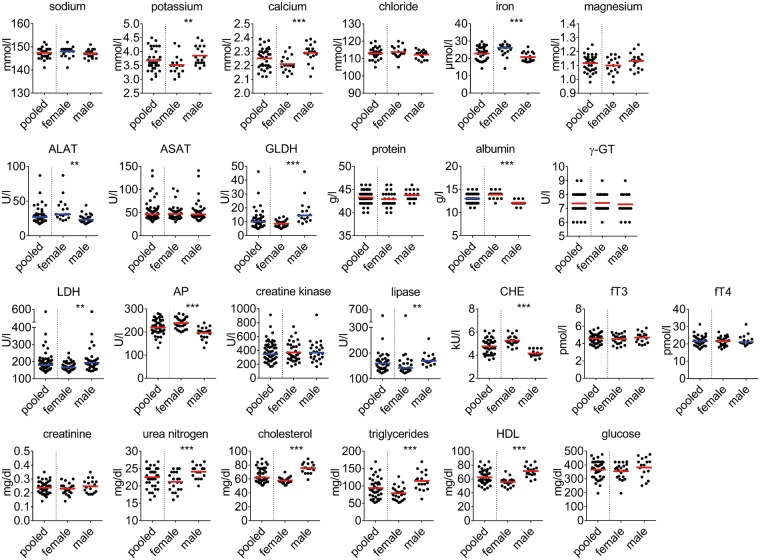
Clinical chemistry parameters. Each data point represents one animal. Lines indicate mean (in red) or median (in blue) for normally distributed or non-normally distributed populations, respectively. Data was pooled from n = 35–73 animals, derived from a total of six independent measurements. Stars indicate statistical significance.

**Figure 2:**
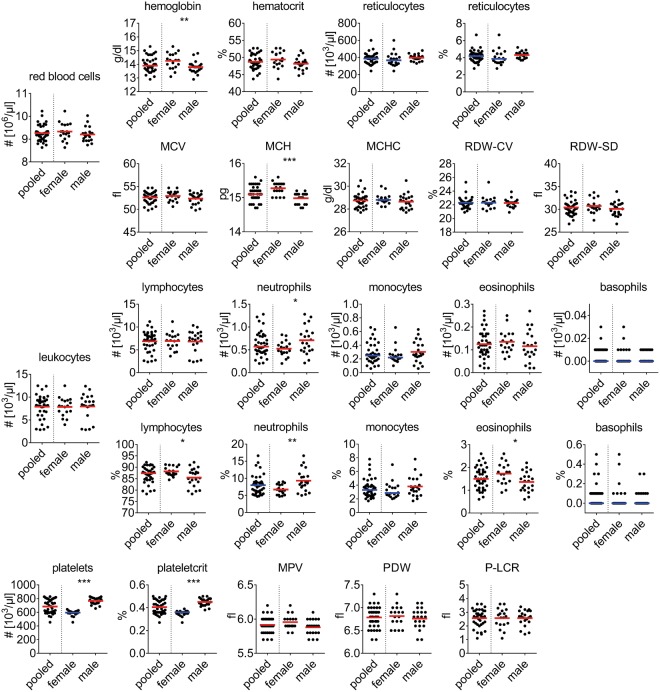
Hematological parameters. Each data point represents one animal. Lines indicate mean (in red) or median (in blue) for normally distributed or non-normally distributed populations, respectively. MCV, mean corpuscular volume; MCH, mean cellular hemoglobin; MCHC, mean corpuscular/cellular hemoglobin concentration; RDW-CV, red blood cell distribution width - coefficient of variation; RDW-SD, red blood cell distribution width - standard deviation; MPV, mean platelet volume; PDW, platelet distribution width; P-LCR, platelet large cell ratio. Data was pooled from n = 39 animals acquired in four independent measurements. Stars indicate statistical significance.

To determine sex-dependent reference intervals for each of the 51 parameters we sequentially analyzed blood of multiple littermates and pooled the data for further analysis. We utilized the robust reference interval method for calculation as the number of animals examined was below 120, some data sets were non-normally distributed, and/or contained outliers^12,13^. Results of clinical chemistry parameters are shown in Fig. 1 and Table 1. Plasma concentration of several electrolytes (calcium, iron, and potassium), enzyme activities (ALAT, aspartate aminotransferase (ASAT), glutamate dehydrogenase (GLDH), LDH, lipase, and pseudocholinesterase (CHE)), albumin, cholesterol, triglycerides, high density lipoprotein (HDL), and urea nitrogen were significantly different between female and male mice. Results of differential blood counts are shown in Fig. 2 and Table 2. We found significant sex differences for values of hemoglobin, mean cellular hemoglobin (MCH), platelets, plateletcrit and minor but statistically significant differences for absolute cell counts of neutrophils, and relative distribution of lymphocytes, neutrophils and eosinophils.

Next, to compare our results to those published by other authors we focused on reports on blood analyses of eight to twelve week old C57BL/6 mice. Table 3 lists the means or medians of laboratory tests as they have been reported by five different laboratories^14–18^ (Table 3). We found most of the values to be comparable to the data acquired in the current study. However, our measurements of blood glucose concentration were approximately two to three-fold higher and those of albumin two to three-fold lower than the values reported by all other laboratories. Our dataset did not allow for meaningful analyses of age-related differences in blood. However, to allow a comparative analysis of this study to results obtained by other laboratories we aim to upload our data to the mouse phenome database (phenome.jax.org)^14^.

**Table 3:** Overview of laboratory test values published in comparison to the present study.

Parameter	Unit	Sex	Present study	Jax.org^57^	Klempt *et al*.^17^	Champy *et al*.^15^	Boehm *et al*.^18^	Zhou *et al*.^16^
ALAT	U/l	f	31	43	14	34	22	27
		m	23	57	15	33	21	27
albumin	g/l	f	14	39				30
		m	12	37				30
AP	U/l	f	239		239	154	129	
		m	197		178	151	153	
ASAT	U/l	f	47		32	91	51	43
		m	46		32	75	45	32
calcium	mmol/l	f	2.21	2.65	2.20	2.37	2.06	
		m	2.29	2.60	2.10	2.45	2.04	
chloride	mmol/l	f	114			116		
		m	112			114		
cholesterol	mg/dl	f	58	79	81	70	89	78
		m	76	100	93	96	77	112
creatinine	mg/dl	f	0.23		0.28	0.34	0.17	0.19
		m	0.25		0.30	0.36	0.19	0.20
creatine kinase	U/l	f	370	720	77	406	102	
		m	367	799	104	571	139	
glucose	mg/dl	f	355	176	88	115	178	106
		m	382	156	108	124	150	157
HDL	mg/dl	f	56	67		56		53
		m	71	90		71		83
iron	µmol/l	f	26.1			19.0		
		m	20.8			16.0		
LDH	U/l	f	167			429	293	
		m	194			453	348	
magnesium	mmol/l	f	1.10			1.13		
		m	1.13			1.25		
potassium	mmol/l	f	3.5			4.8	4.6	5.8
		m	3.9			5.1	4.3	6.4
protein	g/l	f	43	61	53	51	40	47
		m	44	60	53	52	40	49
sodium	mmol/l	f	148			152	158	156
		m	147			151	155	151
triglycerides	mg/dl	f	79	93	109	85	97	69
		m	114	80	97	110	97	83
urea nitrogen	mg/dl	f	21	27	76	31	24	
			24	24	72	35	25	
leukocytes	10^3^/µl	f	7.9	3.5	4.0	7.2		
		m	7.9	2.6	3.4	8.2		
red blood cells	10^6^/µl	f	9.3	10.8	9.1	9.5		
		m	9.2	10.6	9.1	9.9		
hemoglobin	g/dl	f	14.3	17.0	13.6	14.0		
		m	13.8	16.2	13.2	15.0		
hematocrit	%	f	49.4	51.5		43.0		
		m	48.2	52.1		45.0		
MCV	fl	f	52.9	47.8	49.0	45.0		
		m	52.3	49.2	48.6	45.0		
MCH	pg	f	15.3	15.8		15.0		
		m	15.0	15.4		15.0		
MCHC	g/dl	f	28.8	33.2		34.0		
		m	28.6	31.2		33.0		
platelets	10^3^/µl	f	595	1019	518	1138		
		m	767	1157	633	1305		

Means^14,16,17^ or medians^18^ of each parameter are depicted. Values were converted into units as depicted and rounded up if applicable. The animals were analyzed at the age of 8–12 weeks, sample sizes ranged from n = 20 to n = 139. f = female; m = male.

### Laboratory tests for detection of inter-strain differences

**Figure 3:**
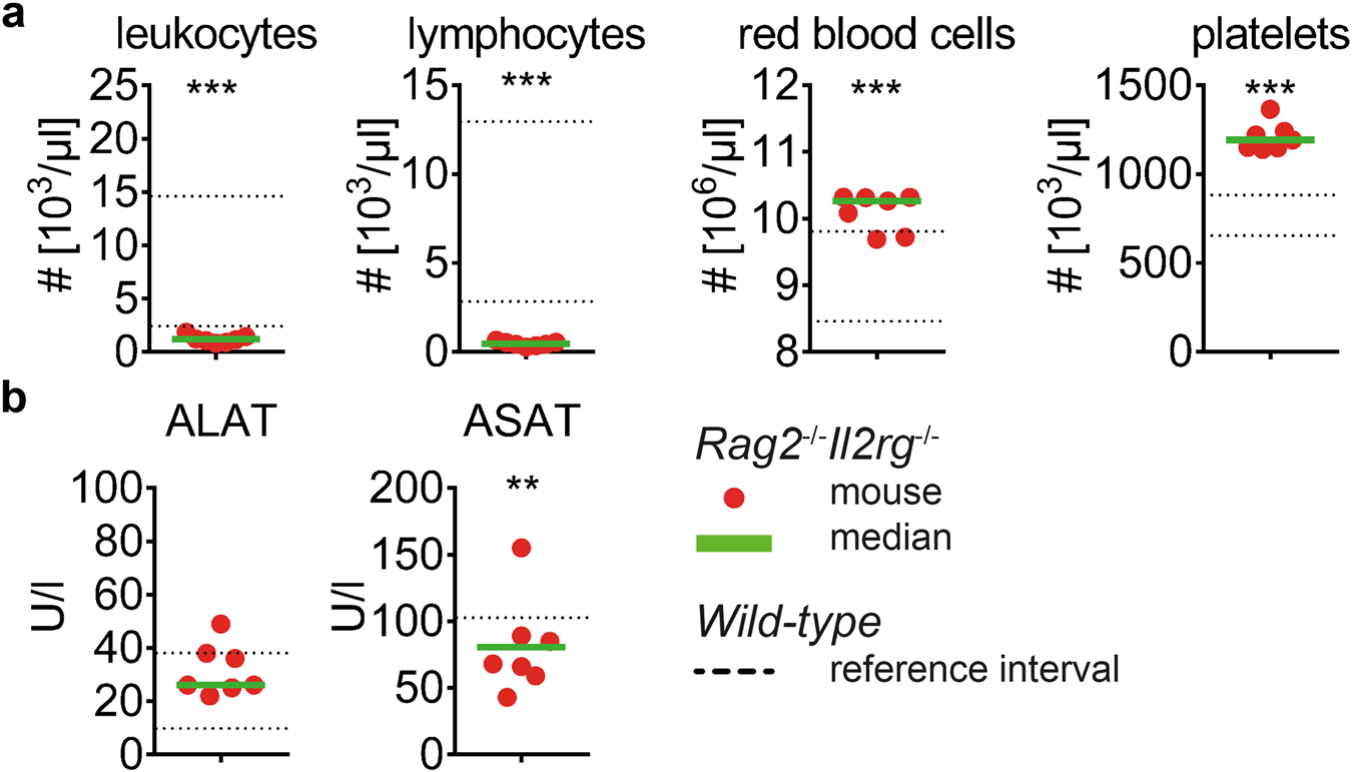
Comparative analysis of blood composition of *wild-type* and *Rag2*^−/−^*Il2rg*^−/−^ mice. Blood composition of *Rag2*^−/−^*Il2rg*^−/−^ male mice was analyzed and depicted in comparison to reference intervals of *wild-ty*pe mice as defined in this study. (**a**) Blood cell counts; (**b**) liver enzymes. Each data point represents one animal, green lines indicate median, dotted lines indicate upper and lower limits of *wild-type* reference intervals as depicted in Tables 1 and 2 (lower border of ASAT is 0). Data was pooled from n = 7 animals of two independent measurements. The non-parametric Mann-Whitney test was used to test for statistical difference between values obtained from *Rag2*^−/−^*Il2rg*^−/−^ mice and those obtained from *wild-type* mice for reference interval calculation.

After evaluation of *wild-type* C57BL/6J mice, we aimed to determine the blood composition of a genetically modified mouse strain. We analyzed peripheral blood of *Rag2*^−/−^*Il2rg*^−/−^ mice in which the targeted mutations interfere with development of T, B and NK cells. Indeed, due to a distinct reduction of lymphocytes the total number of leukocytes in peripheral blood was decreased if compared to *wild-type* mice (Fig. 3a). The number of monocytes and granulocytes seemed not to be affected by this genetic modification (data not shown) and the number of red blood cells was within or slightly above the reference interval (10.3 vs. 9.2 [×10^6^/µl], *Rag2*^−/−^*Il2rg*^−/−^ median vs. *wild-type* mean). Notably, *Rag2*^−/−^*Il2rg*^−/−^ exhibited an increased number of platelets in peripheral blood (1192 vs. 767 [×10^3^/µl], *Rag2*^−/−^*Il2rg*^−/−^ median vs. *wild-type* mean). Enzymatic activity of ALAT and ASAT (68 vs. 46 [U/l], *Rag2*^−/−^*Il2rg*^−/−^ median vs. *wild-type* mean) was comparable to that in plasma of *wild-type* mice (Fig. 3b). In summary, *Rag2*^−/−^*Il2rg*^−/−^ mice exhibited a distinct decrease of lymphocytes in peripheral blood whereas concentrations of liver enzymes were similar to *wild-type* mice.

### Laboratory tests for diagnosis of MCMV-induced liver damage

**Figure 4:**
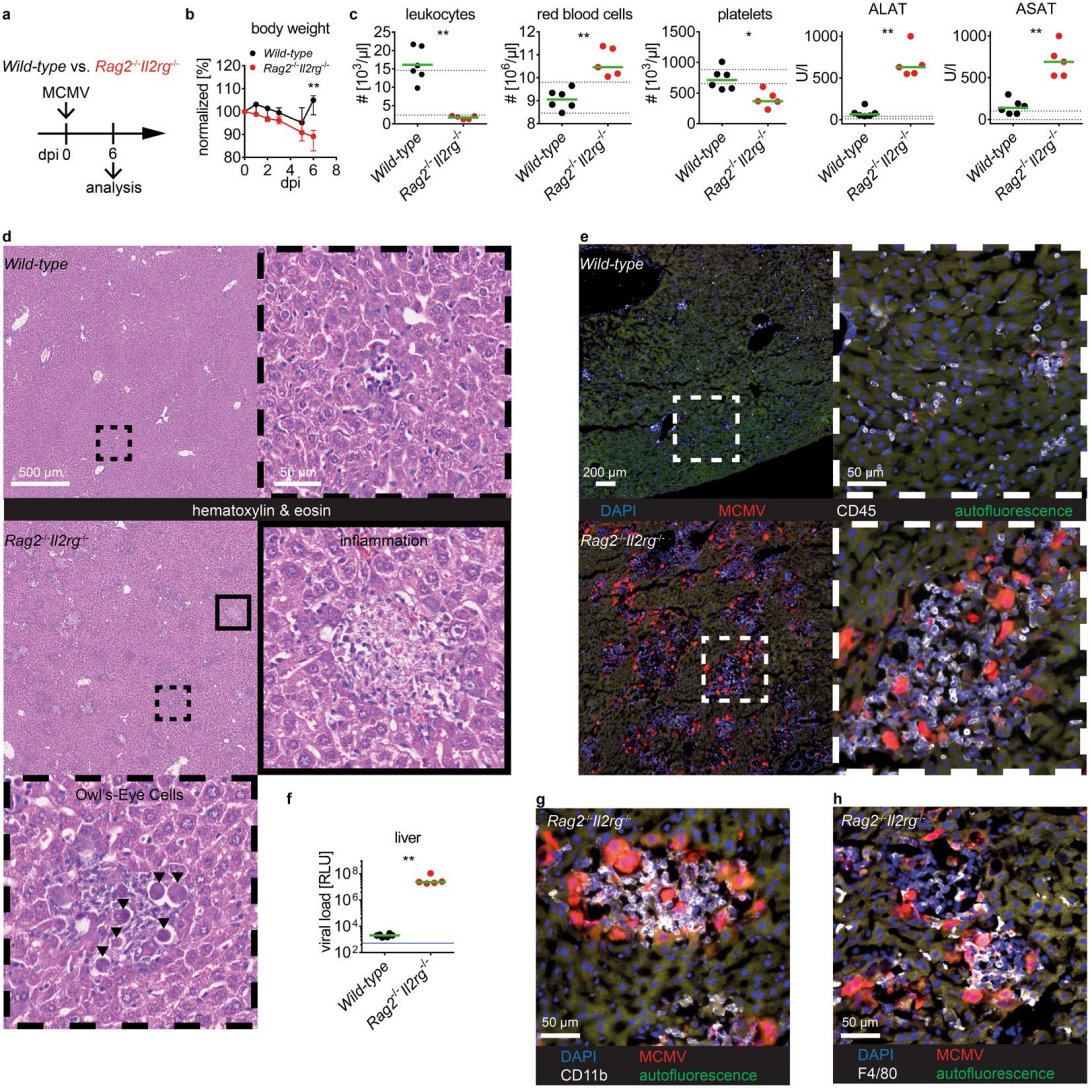
Clinical pathology for diagnosis of MCMV disease. *Wild-type* or *Rag2*^−/−^*Il2rg*^−/−^ male mice were intravenously infected with 10^6^ PFU MCMV and analyzed 6 dpi. (**a**) Experimental setup; (**b**) body weight curves; (**c**) cell counts and plasma activity of ALAT and ASAT; (**d**) representative HE- stained liver sections, (**e**) and detection of MCMV-encoded mCherry in liver cryosections stained for CD45, framed items show higher magnification of the anatomic region as indicated in the overview images, (**f**) viral loads determined by quantification of MCMV-encoded *Gaussia* luciferase activity; cryosections of livers stained for **g**) CD11b and (**h**) F4/80. (**b**) Median and interquartile range, (**c**) and (**f**) each data point represents one animal, green lines indicate median, dotted lines indicate upper and lower limits of *wild-type* reference intervals as depicted in Tables 1 and 2, (**f**) blue line indicates detection limit of the assay. Scale bars: (**d**) 500 µm and 50 µm for low and high magnification, respectively; (**e**) 200 µm and 50 µm for low and high magnification, respectively. (**g**) and (**h**) 50 µm. Data was pooled from n = 5–6 animals per group, analyzed in two independent experiments, dpi = days post infection, RLU = relative light units. Stars indicate statistical significance.

Next, we aimed to evaluate the use of laboratory tests in a murine disease model. For that, we utilized MCMV as a model pathogen to induce infectious disease in mice. We intravenously infected *Rag2*^−/−^*Il2rg*^−/−^ or *wild-type* mice with a MCMV recombinant that contains the reporter genes mCherry and *Gaussia* luciferase allowing for visualization of infected cells and quantification of virus load, respectively (Fig. 4a). At six days post infection (dpi) *Rag2*^−/−^*Il2rg*^−/−^ mice had lost significantly more body weight as compared to *wild-type* mice indicating an illness caused by virus infection in these animals (Fig. 4b). We applied a small panel of clinical chemistry parameters and a differential blood count to examine potential effects of MCMV infection on blood composition (Fig. 4c). The number of leukocytes in MCMV-infected *wild-type* mice was slightly increased which was due to elevated numbers in monocytes (Fig. 4c and data not shown). However, numbers of red blood cells and platelets were not affected in *wild-type* mice. Likewise, numbers of red blood cells in *Rag2*^−/−^*Il2rg*^−/−^ mice were not affected by MCMV infection. In contrast, MCMV-infected *Rag2*^−/−^*Il2rg*^−/−^ mice showed an approximately two-fold reduction in platelets as compared to non-infected animals (compare Fig. 4c to Fig. 3a). Indeed, thrombocytopenia is a typical feature of acute cytomegalovirus infection in human patients and here we could observe this phenomenon in *Rag2*^−/−^*Il2rg*^−/−^ mice.

As MCMV is known to show tissue tropism to the liver we analyzed concentrations of ALAT and ASAT in peripheral blood. These aminotransferases are found at high concentrations in liver parenchymal cells and are released into blood circulation during cell damage. Thus, both parameters are sensitive indicators of acute liver cell damage. Indeed, in plasma samples of *Rag2*^−/−^*Il2rg*^−/−^, but not of *wild-type* mice, the activities of both enzymes were found to be approximately ten-fold increased to the upper bound of the reference intervals of *wild-type* mice (Fig. 4c). Hence, we sacrificed the animals and performed in-depth examination of the livers. In *wild-type* mice we hardly detected any morphological changes in liver anatomy (Fig. 4d). In contrast, in the livers of *Rag2*^−/−^*Il2rg*^−/−^ mice we found multiple areas of focal inflammation, which contained distinctive enlarged cells with intra-nuclear inclusion bodies (Fig. 4d). These cells with owl’s eyes appearance are typically found in CMV-infected patients as a result of the virus-induced cytopathic effect (CPE)^19^. We confirmed the presence of multiple mCherry^+^ infected cells in the livers co-localizing with an infiltrate of CD45^+^ hematopoietic cells (Fig. 4e). Accordingly, the viral loads were remarkably elevated in livers of *Rag2*^−/−^*Il2rg*^−/−^ mice whereas *wild-type* mice had sufficiently controlled the virus infection at six dpi (Fig. 4f). The immune cells present at the site of infection were positive for CD11b, which is an indicative cell surface marker for cells of the myeloid lineage (Fig. 4g). In addition, we identified F4/80^+^ macrophages (Fig. 4h). Thus, in *Rag2*^−/−^*Il2rg*^−/−^ mice MCMV infection caused severe liver damage with increased plasma levels of ALAT and ASAT and histopathological findings consistent with virus-induced hepatitis. Hence, we could detect organ damage with tissue-specificity in an experimental infection model by using blood-based laboratory diagnostic tests.

### Plasma activities of aminotransferases correlate with viral loads in livers

**Figure 5:**
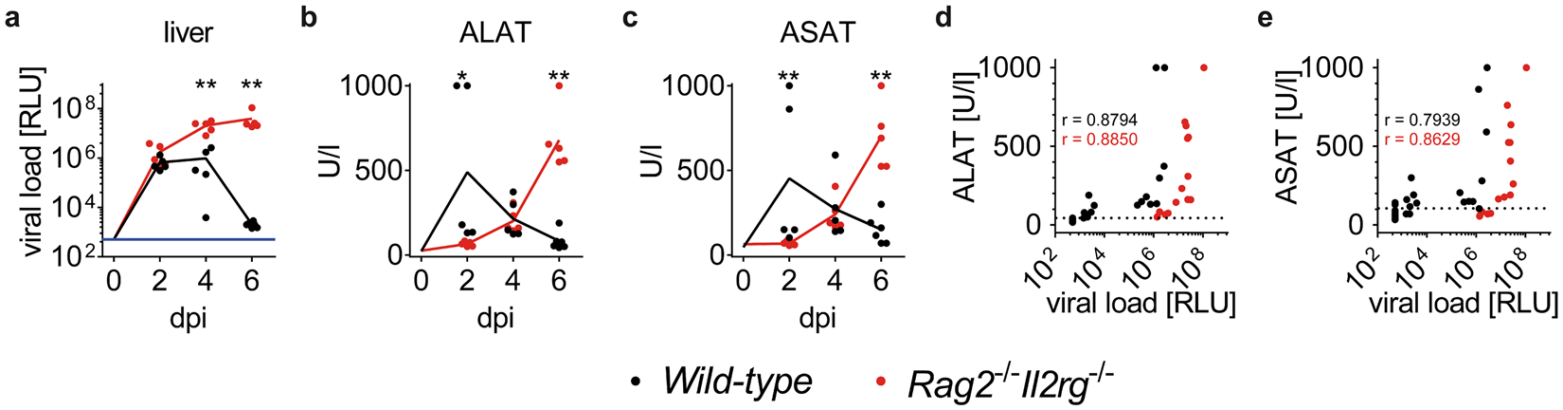
Concentration of plasma aminotransferases and viral loads in acute MCMV infection. *Wild-type* or *Rag2*^−/−^*Il2rg*^−/−^ male mice were intravenously infected with 10^6^ PFU MCMV and analyzed at various days post infection. (**a**) Viral loads in livers at dpi as indicated, the value at 0 dpi was set to detection limit of the assay to allow visualization of kinetics, data for six dpi is depicted as in Fig. 4f, blue line indicates detection limit of the assay; plasma activity of (**b**) ALAT and (**c**) ASAT at dpi as indicated, the value at 0 dpi was set to median of values measured in untreated mice to allow visualization of kinetics, data for six dpi is depicted as in Fig. 4c; plasma activity of (**d**) ALAT and (**e**) ASAT in correlation to viral loads in liver, data from non-infected animals were included and RLU values set to detection limit of the assay. Data was pooled from n = 5–6 animals per group, analyzed in two independent experiments, dpi = days post infection, RLU = relative light units, r = Spearman coefficient. Stars indicate statistical significance.

Finally, we investigated if ALAT and ASAT plasma levels were applicable to detect MCMV-induced hepatitis early after infection. Therefore, we analyzed intravenously infected *Rag2*^−/−^*Il2rg*^−/−^ or *wild-type* mice already at two and four dpi for viral loads in the liver and concentration of the two enzymes in peripheral blood. *Wild-type* mice showed similar viral loads at two dpi as observed in *Rag2*^−/−^*Il2rg*^−/−^ mice (Fig. 5a). However, at four dpi the viral loads were significantly higher in *Rag2*^−/−^*Il2rg*^−/−^ mice whereas some *wild-type* mice already had lower viral loads than observed at two dpi. This difference was more distinctive at six dpi indicating that *wild-type* mice are capable of controlling the infection within this period whereas viral loads further increased in *Rag2*^−/−^*Il2rg*^−/−^ mice. In line with these data, blood concentrations of ALAT and ASAT increased over time in *Rag2*^−/−^*Il2rg*^−/−^ mice (Fig. 5b+ c). In contrast, blood concentrations of liver enzymes showed a less distinctive pattern in *wild-type* mice. However, at two dpi, both enzymes showed higher activity in *wild-type* than in *Rag2*^−/−^*Il2rg*^−/−^ mice indicating that increased cell damage was present in *wild-type* mice at this time point. Thus, different pathophysiological mechanisms caused the release of aminotransferases from hepatocytes in the two animal models.

Plasma concentrations of ALAT as well as ASAT correlated with viral loads in liver of *wild-type* and *Rag2*^−/−^*Il2rg*^−/−^ mice (Fig. 5d+ e). Moreover, ALAT plasma activity was found in 100% (16/16) of *wild-type* and 100% (15/15) of *Rag2*^−/−^*Il2rg*^−/−^ mice to be above the upper limit of the reference interval. 87.5% (14/16) of *wild-type* and 66.7% (10/15) of *Rag2*^−/−^*Il2rg*^−/−^ mice had ASAT levels above the reference interval. Thus, ALAT appeared to be a sensitive parameter to detect MCMV infection of livers in immune-competent and lymphopenic mice.

## Discussion

The use of animal models in life sciences is an established means to study pathophysiology of various diseases *in vivo*. Body weight, food and water intake, fur and skin conditions, physical activity, behavior, and the mouse grimace scale are frequently assessed by scientists to monitor the health status of experimental mice. Although these parameters can be determined via non-invasive procedures some of them are observer-dependent and all of them lack specificity for a certain disease. In contrast, the use of laboratory diagnostics from a single blood withdrawal may provide detection of pathophysiological processes with tissue specificity. In the present study, we generated reference intervals of human laboratory tests for mice that allow for detection of tissue damage in various organs including the hematopoietic system, liver, kidney, pancreas, skeletal muscle, and others. We illustrate how these diagnostic methods can be applied to detect virus-induced hepatitis in MCMV-infected *Rag2*^−/−^*Il2rg*^−/−^ mice indicating that the observed body weight loss in these animals is, at least in part, due to liver inflammation.

Although we are not the first to investigate the use of laboratory diagnostics for murine samples, we here present an elaborate panel of parameters and used a substantial number of mice to determine reference intervals. In this study, we focused on the C57BL/6J mouse, one of the most widely used inbred strain, to allow for adaptation and comparison of the obtained data to genetically modified mice backcrossed to the same strain. Although others have reported measurements of laboratory tests for C57BL/6 mouse blood we could not adapt their confidence intervals to our platform. Comparison of values between different laboratories is limited in general as there are diverging detection methods and reagents for each individual parameter. Indeed, some parameters such as bilirubin that was investigated by others^20^ could not be measured reliably on our analyzers. Likewise, differential blood count measurements could not be processed on our human hematology analyzers. Instead, we processed the mouse blood with a veterinarian analyzer that provided species-specific cell gating strategies. Furthermore, analysis of animal blood composition may be affected by the various techniques used for blood withdrawal^21–23^ or the relative volume of anti-coagulant present in the sampling tube. Finally, various housing conditions influence gut microbiota and likely affect the blood composition of experimental animals^24^. Thus, there are multiple explanations for the observed inter-laboratory differences and we cannot provide a specific explanation for the aberrant values measured for albumin or glucose in this study. However, the measured blood glucose concentrations appeared non-physiological and therefore we do not recommend using this test in future studies.

The sex-dependent differences for blood cells and some clinical chemistry parameters observed in the present study have not consistently been reported by others. Accordingly, we could not find any reports explaining the observed higher LDH plasma concentrations in male mice. Differences in cholesterol and HDL levels between female and male mice have been described previously^25,26^.

MCMV has a broad tissue tropism and MCMV-associated hepatitis has been described in various mouse strains via histological analysis and plasma activity of aminotransferases^27–34^. Viral loads are high in livers of MCMV-infected mice within the first four dpi and then decline within one week. In line with the high viral loads plasma activity of liver enzymes is increased early after infection and reduction of enzyme activity is paralleled by the drop of viral titers after intraperitoneal application of the virus^29,30^. As immune control of MCMV infection relies on T and NK cells^5,6^, higher viral loads can be found in organs at six dpi in *Rag2*^−/−^*Il2rg*^−/−^ as compared to *wild-type* mice. Likely, control of MCMV infection by lymphocytes in immune-competent mice leads to early liver cell damage and release of aminotransferases into peripheral blood. Thus, plasma activity of ALAT and ASAT are increased in *wild-type* mice and drop until six dpi when most of infected cells are controlled by the immune system. In contrast, lymphopenic mice allow ongoing replication of MCMV and the release of liver enzymes is rather due to virus-mediated CPE than by an immune cell-mediated mechanism. Virus-mediated CPE occurs later after infection as plasma concentrations of ALAT and ASAT were higher in *wild-type* than in *Rag2*^−/−^*Il2rg*^−/−^ mice at two dpi. Similar findings were observed in other immune-compromised (SCID) or T cell-depleted mice where immunopathology was less pronounced than in *wild-type* controls at four dpi^35^. A progressive MCMV hepatitis has also been observed in T and NK cell-deficient E26 mice^29^. In general, mice lacking T, B and/or NK cells or irradiated mice show higher viral loads, morbidity and mortality than immune-competent animals^36–39^. However, due to the various antiviral mechanisms mediated by different immune cell types these phenotypes may occur early or late after infection. To our knowledge the present study is the first to report MCMV-associated hepatitis in *Rag2*^−/−^*Il2rg*^−/−^ mice. As NK and T cells are crucial to control the acute MCMV infection phase^5,6,39,40^ this animal model is equivalent to other models with impaired innate and adaptive immunity. The presence of multiple juxtapositioned MCMV-infected cells and a local immune cell infiltrate show morphological similarities to nodular inflammatory foci (NIFs) which are found in immunocompromised patients infected with human cytomegalovirus^41^, have previously been described in lungs of neonatal and adult *wild-type* mice^42^, and similar structures have been also observed in various organs and mouse models^27,28,35,43–45^. Interestingly, lymphocytes are not a prerequisite for the formation of NIFs as myeloid cells accumulate at the site of infection independently from the presence of T and NK cells^39^. In contrast, plaque-like formation of infected cells without immune cell infiltrates have been observed in gamma-irradiated mice which lack both myeloid and lymphoid cells^36,39,46,47^.

As *Rag2*^−/−^*Il2rg*^−/−^ mice exhibit a distinctive lymphopenia we stained liver sections for CD11b and F4/80 which are typically expressed by myeloid cells. Interestingly, absolute numbers of monocytes were decreased in peripheral blood of MCMV-infected *Rag2*^−/−^*Il2rg*^−/−^ mice (data not shown) indicating that either many of the cells were captured in the liver or MCMV interfered with hematopoiesis. Since also the number of platelets was also decreased after MCMV infection impaired hematopoiesis appears to be more likely^48–50^. In the absence of T and NK cells, MCMV leads to severe liver cell damage at six dpi as plasma activities of aminotransferases were considerably increased in *Rag2*^−/−^*Il2rg*^−/−^ mice. This data argues that the observed tissue lesions can occur without the presence of lymphocytes. Thus, potential roles of (i) direct CPE of MCMV and (ii) myeloid cells present in NIFs in tissue damage of MCMV-infected mice need to be considered in future studies.

The use of reference intervals allowed for discrimination of MCMV-infected from non-infected *wild-type* and *Rag2*^−/−^*Il2rg*^−/−^ mice as ALAT plasma concentrations were above the upper limit of the reference values in all infected animals tested. Thus, ALAT is a sensitive marker for detection of MCMV-mediated liver damage in experimental animals. Moreover, viral loads in livers as well as plasma levels of ALAT and ASAT showed comparable kinetics in *Rag2*^−/−^*Il2rg*^−/−^ mice. Accordingly, enzyme activities >300 U/l likely correlate with viral disease that may indicate suffering of the animals as values >500 U/l were only found in animals with very high viral loads and pronounced liver pathology at six dpi. Thus, aminotransferases are suitable parameters to monitor the health status of MCMV-infected *Rag2*^−/−^*Il2rg*^−/−^ mice.

Taken together, the use of laboratory diagnostics allows for rapid detection and monitoring of infection-induced liver damage. Moreover, utilizing laboratory diagnostics for early detection of MCMV disease will allow us to promptly terminate an experiment and minimize pain and suffering of the animal to improve animal welfare (refinement). Moreover, the use of highly quality-controlled methods reduces the variance of values and therefore the number of mice needed to characterize robust phenotypes in mouse models (reduction). Hence, application of the methods described here are in line with principles of the 3 R’s (Replacement, Reduction and Refinement) of animal welfare. We offer the screeing service to others (www.hheld.net).

In summary, we established reference intervals for important clinical pathology laboratory tests and illustrated several sex differences for C57BL/6J mice. Application of laboratory diagnostics in peripheral blood of experimental mice allowed for organ-specific detection of disease. This diagnostic platform complements other methods to monitor the health status of experimental animals.

## Methods

### Animals

C57BL/6J mice were purchased from Charles River Laboratories (Sulzfeld, Germany) to generate a colony at the local animal facility of the University Medical Center Hamburg-Eppendorf. Mice were bread in individually ventilated cages under specific pathogen free conditions according to the recommendations of the Federation of European Laboratory Animal Science Associations (FELASA)^51^. *Pasteurella pneumotropica*, *Helicobacter* species and murine norovirus 1 were detected in sentinel animals tested in this breeding barrier. Food and water was provided *ad libitum*. Animals were analyzed at the age of 6–10 weeks, *Rag2*^−/−^*Il2rg*^−/−^^52,53^ were on C57BL/6 background and maintained similarly.

### Ethics statement

All animal experiments were performed according to the recommendations and guidelines of the FELASA and Society of Laboratory Animals (GV-SOLAS) and approved by the institutional review board and local authorities (Behörde für Gesundheit und Verbraucherschutz, Amt für Verbraucherschutz, Freie und Hansestadt Hamburg, reference numbers 85/15 and 39/17).

### Blood withdrawal

Blood was obtained from the retro-orbital plexus with the use of heparinized micro-haematocrit tubes (Vitrex, Product No. 161813) in deep anesthesia (ketamine and xylazine). Blood was subsequently collected in tubes prefilled with EDTA (Kabe Labortechnik GmbH, Product No. 077011) for determination of differential blood counts or heparin (Sarstedt AG & Co, Product No. 41.1393.005) for determination of clinical chemistry parameters, respectively.

### Clinical Chemistry

Heparin-anticoagulated plasma was obtained following 10 min of centrifugation at 2000 rcf and analyzed for clinical chemistry parameters on Dimension Vista 1500 Lab Systems (Siemens Healthcare) at the Central Laboratory of the University Medical Center Hamburg-Eppendorf within four hours after blood withdrawal. Sodium, potassium and chloride were quantified by indirect potentiometry; calcium, iron, magnesium, total protein, albumin, creatinine, urea-nitrogen, cholesterol, triglycerides, HDL and glucose as well as enzymatic activity of ALAT, ASAT, LDH, gamma-glutamyl transpeptidase (ɣ-GT), alkaline phosphatase (AP), creatine kinase, lipase, CHE were quantified photometrically with reagents provided by Siemens Healthcare. The hormones free triiodothyronine (fT3) and free tyroxine (fT4) were quantified using a Luminescent Oxygen Channeling Immunoassay. GLDH was assessed using the reagents from Roche Diagnostics. All procedures were performed according to the manufacturers’ protocols.

### Hematology

Differential blood counts were analyzed from EDTA-anticoagulated whole blood samples using a ProCyte Dx Hematology Analyzer (IDEXX Laboratories) within four hours after blood withdrawal. Briefly, this system uses a combination of optical fluorescence, laminar flow impedance, and cyanide-free sodium lauryl sulphate (SLS) - hemoglobin method to determine all presented parameters in one run.

### Statistical Analysis

We performed a d’Agostino and Pearson omnibus normality test for each analyte to test for Gaussian distribution. Accordingly, the parametric unpaired Student’s t-test was used for normally distributed whereas the non-parametric Mann-Whitney test was used for non-normally distributed populations to compare results obtained from female or male mice. The non-parametric (Spearman) test was performed to analyze for correlation of viral loads and liver enzymes. The mean or median is given for normally distributed or non-normally distributed populations, respectively. Statistical significance was depicted as follows: *p < 0.05; **p < 0.01; and ***p < 0.001. Data was processed with GraphPad Prism (version 6) software.

Reference intervals were determined by the two-sided 95% robust reference interval according to Horn *et al*.^13^ with the following constants: tuning constant 1 = 3.700, tuning constant 2 = 205.408, MAD scale factor = 0.674500, and bootstrap samples = 10000. Data was processed with NCSS statistical software (version 11.0.13).

### MCMV infection

The MCMV-3DR recombinant has been described previously^54–56^ and was produced on 10.1 immortalized mouse embryonic fibroblasts^45^ and titrated on M2-10B4 mouse bone marrow stromal cells (ATCC CRL-1972). MCMV-3DR was generated from the pSM3fr bacterial artificial chromosome. It encodes *Gaussia* luciferase, *mCherry*, contains a sequence within the *m164* ORF encoding the SIINFEKL peptide, and contains the complete *Mck2* ORF but its *m157* ORF is replaced by the sequences for the reporter proteins. Mice were anaesthetized (isoflurane) and received a single intravenous injection of 10^6^ PFU MCMV-3DR.

### Histology

Organs were fixed in either PBS-buffered 2% paraformaldehyde with 30% sucrose for cryosections and immune fluorescence histology, or 4% formalin for paraffin sections and hematoxylin & eosin (HE) staining. For immune fluorescence histology 7 µm-thick organ slices were stained with antibodies (clone) after serum blocking as follows: CD45-APC (30-F11), CD11b-APC (M1/70), or F4/80-APC (BM8). Images were taken with an Axiovert 200 M fluorescence microscope (Carl Zeiss) and processed with AxioVision 4.9 software. Images of HE stained sections were generated by the local mouse pathology facility.

### Luciferase assay

Explanted livers were kept on ice-cooled saline, homogenized with TissueLyser II (Qiagen), centrifuged, and supernatants were measured for luciferase expression by quantification of luminescence after the addition of native coelenterazine (Synchem) with Centro XS³ LB 960 (Berthold Technologies).

## Electronic supplementary material

Supplementary Information

## Data Availability

Data obtained for calculation of reference intervals will be uploaded to the mouse phenome database (phenome.jax.org)^14^.
