# Supplementary material for: Laboratory diagnostics of murine blood for detection of mouse cytomegalovirus (MCMV)-induced hepatitis
Source: Sci Rep. 2018 Oct 4;8:14823. doi: 10.1038/s41598-018-33167-7 (PMC6172243; doi:10.1038/s41598-018-33167-7)
Supplement: Supplementary file 1 — Supplementary Information [file 41598_2018_33167_MOESM1_ESM.docx]

**Laboratory diagnostics of murine blood for detection of mouse cytomegalovirus (MCMV)-induced hepatitis**

Felix R. Stahl, Roman Jung, Virginija Jazbutyte, Eléonore Ostermann, Silvia Tödter, Renke Brixel, Annette Kemmer, Stephan Halle, HallhStefan Rose-John, Martin Messerle, Petra C. Arck, Wolfram Brune, Thomas Renné

**Supplementary information Table 1**

| **Parameters that could not be measured** | **Parameters not further analysed because large plasma volume needed per measurement** |
| --- | --- |
| alpha feto protein | dehydroepiandrosterone sulfate |
| amylase | estrogen |
| beta 2 microglobulin | parathormone |
| beta-human choriongonadotropin | progesterone |
| bilirubin direct | testosterone |
| bilirubin total | vitamin D |
| complement 3 |  |
| complement 4 |  |
| C-reactive protein |  |
| creatine kinase MB |  |
| creatine kinase MB mass |  |
| cystatin C |  |
| ferritin |  |
| follicle-stimulating hormone |  |
| haptoglobin |  |
| hemopexin |  |
| high sensitive C-reactive protein |  |
| homocysteine |  |
| insulin |  |
| Interleukin 6 |  |
| luteinizing hormone |  |
| myoglobin |  |
| N-terminal fragment of pro-BNP |  |
| phosphor |  |
| proinsulin C peptides |  |
| prolactin |  |
| thyroid-stimulating hormone |  |
| transferrin |  |
| uric acid |  |
| vitamin B12 |  |

List of parameters that were not further analyzed due to technical limitations as assigned.
